# Losing the Battle but Winning the War: Can Defeated Antibacterials Form Alliances to Combat Drug-Resistant Pathogens?

**DOI:** 10.3390/antibiotics10060646

**Published:** 2021-05-28

**Authors:** Song Oh, Raymond Chau, Anh T. Nguyen, Justin R. Lenhard

**Affiliations:** Department of Clinical and Administrative Sciences, College of Pharmacy, California Northstate University, Elk Grove, CA 95757, USA; Song.Oh@cnsu.edu (S.O.); Raymond.Chau7992@cnsu.edu (R.C.); Anh.Nguyen4390@cnsu.edu (A.T.N.)

**Keywords:** *Staphylococcus aureus*, *Pseudomonas aeruginosa*, *Klebsiella pneumoniae*, carbapenem resistance, *Acinetobacter baumannii*, antimicrobial combinations

## Abstract

Despite the recent development of antibacterials that are active against multidrug-resistant pathogens, drug combinations are often necessary to optimize the killing of difficult-to-treat organisms. Antimicrobial combinations typically are composed of multiple agents that are active against the target organism; however, many studies have investigated the potential utility of combinations that consist of one or more antibacterials that individually are incapable of killing the relevant pathogen. The current review summarizes in vitro, in vivo, and clinical studies that evaluate combinations that include at least one drug that is not active individually against *Pseudomonas aeruginosa*, *Klebsiella pneumoniae*, *Acinetobacter baumannii*, or *Staphylococcus aureus*. Polymyxins were often included in combinations against all three of the Gram-negative pathogens, and carbapenems were commonly incorporated into combinations against *K. pneumoniae* and *A. baumannii*. Minocycline, sulbactam, and rifampin were also frequently investigated in combinations against *A. baumannii*, whereas the addition of ceftaroline or another β-lactam to vancomycin or daptomycin showed promise against *S. aureus* with reduced susceptibility to vancomycin or daptomycin. Although additional clinical studies are needed to define the optimal combination against specific drug-resistant pathogens, the large amount of in vitro and in vivo studies available in the literature may provide some guidance on the rational design of antibacterial combinations.

## 1. Introduction

The use of antibacterial combinations may be advantageous for the treatment of acute bacterial infection in multiple situations. During empiric therapy, the simultaneous use of multiple antimicrobials may confer a wider spectrum of activity against suspected organisms. Once the pathogenic organism is identified, directed therapies may use multiple antibacterials to prevent the emergence of drug-resistance, enhance killing against difficult-to-treat pathogens, and prevent toxin production. Most antibacterial combinations utilize antimicrobials that individually possess activity against the target organism; however, there may be clinical scenarios in which it is desirable to include an agent into a combination regimen despite the presence of resistance mechanisms that confer non-susceptibility to the antibacterial of interest. 

The current review provides a summary of available literature that investigates the use of antibacterial combinations utilizing at least one antimicrobial that is not individually active against the target pathogen. The three Gram-negative organisms *Pseudomonas aeruginosa*, *Klebsiella pneumoniae*, and *Acinetobacter baumannii*, and the Gram-positive pathogen *Staphylococcus aureus* are each evaluated based on clinically relevant drug resistance profiles. Lastly, the review concludes by commenting on which of the combination regimens offer the most promise for the treatment of specific drug-resistant pathogens.

## 2. Methodology

To focus the discussion of the review, clinically relevant pathogens that frequently possess multidrug-resistant phenotypes were identified by the authors. *P. aeruginosa*, *A. baumannii*, and *Enterobacteriaceae* are Gram-negative organisms that frequently are resistant to carbapenems [1,2], have been identified as urgent or serious threats to public health by the Centers for Disease Control and Prevention [3], and sometimes merit treatment with polymyxins or other last line antibacterials due to extensive drug resistance [4]. Although the family *Enterobacteriaceae* contains many organisms, *K. pneumoniae* carbapenemase-producing *K. pneumoniae* (KPCKP) are one of the most commonly encountered carbapenem-resistant *Enterobacteriaceae* in the world, and *K. pneumoniae* was therefore selected as a representative of the *Enterobacteriaceae* family; however, it should be noted that *Escherichia coli*, *Salmonella* species, and *Shigella* species are clinically relevant organisms that were not included in the current review [5]. *S. aureus* was also included in the review due to its ubiquitous presence in the clinic, status as a serious threat to public health, and the availability of numerous studies that investigated relevant combinations against the pathogen [3,6]. 

After identifying the organisms that were included in the study, multiple searches in Pubmed were conducted that included permutations of the terms “combination,” “non-susceptible,” “seesaw,” “resistant,” “drug-resistant,” “carbapenem-resistant,” “polymyxin-resistant,” and the names of organisms included in the review. In vitro, in vivo, and clinical studies were included in the review if a combination was investigated that utilized at least one agent that was individually not active against the target organisms. Other studies were also included at the discretion of the authors based on referenced works from the original literature search. The antibacterial combinations studied in dynamic in vitro models, in vivo models, and clinical investigations were summarized in a table for each pathogen to highlight the most promising combination regimens. 

## 3. *Pseudomonas aeruginosa*

### 3.1. Multidrug-Resistant P. aeruginosa

Multidrug-resistant (MDR) *P. aeruginosa* is defined as an isolate that is non-susceptible to at least one agent in more than two of the following classes of antipseudomonal drugs: penicillins with beta-lactamase inhibitors, cephalosporins, fluoroquinolones, aztreonam, phosphonic acid, carbapenems, aminoglycosides, and polymyxins [7]. Several combination antimicrobial therapies have been studied to examine the synergistic and additive effects against MDR *P. aeruginosa*. Although rifampin monotherapy is not generally used in *P. aeruginosa* infections, the combination of colistin and rifampin demonstrated synergistic activity in vitro [8,9]. Timurkaynak et al. reported that rifampin and colistin combination therapy demonstrated fully and partially synergistic activity against MDR *P. aeruginosa* strains isolated from intensive care units. All the *P. aeruginosa* strains included in the investigation were resistant to rifampin and several strains were resistant to colistin [8]. Another in vitro checkerboard analysis found that the addition of rifampin to colistin conferred partial synergy in 5/7 strains or full synergy in 1/7 strains of MDR *P. aeruginosa*. Furthermore, colistin and rifampin combination therapy resulted in successful treatment in four patients with sepsis or pneumonia caused by MDR *P. aeruginosa* [9]. 

The addition of amikacin and fosfomycin to non-susceptible beta-lactams or aztreonam was also evaluated as a potential treatment option, although the results are inconsistent. An in vitro study by Oie et al. reported that aztreonam and amikacin effectively inhibited proliferation in 5/7 strains of MDR *P. aeruginosa* that were resistant to ceftazidime, aztreonam, and amikacin [10]. In the time-killing experiments, a triple therapy of ceftazidime, aztreonam, and amikacin achieved synergy at 24 h against 3/7 strains of MDR *P. aeruginosa*. A recently published in vitro study by Mullane et al. evaluated double combinations of either amikacin or fosfomycin together with cefepime, ceftolozane-tazobactam, or meropenem against beta-lactam-resistant *P. aeruginosa*. Most of the combinations demonstrated additive effects against MDR *P. aeruginosa* (53–71%), while meropenem with amikacin or fosfomycin resulted in the least percentage of additivity (30–37%) [11]. Similarly, the combination of ceftolozane-tazobactam with amikacin, colistin, or fosfomycin showed additive and synergistic effects against MDR *P. aeruginosa* strains in both an in vitro dynamic model and time-kill analysis (Table 1) [12,13]. 

Efficacy of polymyxin B with various antibiotics was evaluated in automated time-lapse microscopy and static time-kill experiments. Olsson et al. reported that additive or synergistic activity was observed at 24 h in the time-kill experiments with polymyxin B and aztreonam, cefepime, or meropenem dual combinations [23]. All four strains were MDR *P. aeruginosa* that were intermediate or resistant to polymyxin B. Similar to the previously described synergistic activity achieved with colistin and rifampin, the combination of polymyxin B and rifampin demonstrated synergistic and bactericidal effects against two strains. 

Despite promising results from in vitro studies, evidence supporting the clinical benefit of combination therapy with at least one inactive antimicrobial agent against MDR *P. aeruginosa* is lacking. The majority of clinical studies that investigated antibacterial combinations against MDR *P. aeruginosa* either evaluated combinations that utilized multiple agents that were individually active in vitro, or the susceptibility profiles of the *P. aeruginosa* were not described in the studies [16,17,24,25]. Although a meta-analysis of cohort studies comparing monotherapy and combination therapy for the treatment of *P. aeruginosa* bacteremia reported no significant differences in mortality (Odds Ratio (OR) 0.81, 95% confidence interval (CI) 0.61–1.08), the analysis did not differentiate specific antibiotic therapies and their susceptibility profiles [26]. A systematic review and meta-analysis by Schmid et al. reported lower mortality with combination therapy compared to monotherapy (Relative Risk (RR) 0.83, CI 0.73 to 0.93, *p* = 0.002) in MDR Gram-negative infections [27]. However, the investigators defined combination therapy as two or more antibiotics regardless of in vitro susceptibility, and the subgroup analysis for MDR *P. aeruginosa* showed no mortality benefit with combination therapy (RR 0.95, 95% CI 0.64–1.41; *p* = 0.78).

### 3.2. Extensively Drug-Resistant P. aeruginosa 

A strain of *P. aeruginosa* is considered extensively-drug resistant (XDR) if non-susceptibility is detected in all but two or fewer antipseudomonal drug classes [7]. The current Infectious Diseases Society of America (IDSA) guidance similarly defines extensive drug resistance XDR as “difficult-to-treat” resistance and recommends against routine combination therapy if the isolate demonstrates a confirmed susceptibility to a first-line antibiotic, such as ceftolozane-tazobactam, ceftazidime-avibactam, or imipenem-cilasatin-relebactam [28]. As such, antimicrobial combinations have demonstrated underwhelming in vitro activity against XDR *P. aeruginosa*. Lee et al. evaluated 43 XDR *P. aeruginosa* isolates, which were resistant to all tested antibacterials, except colistin. Combinations including imipenem and colistin, ceftazidime and colistin, and rifampin and colistin exerted only additive or indifferent activity against most XDR *P. aeruginosa* isolates [29]. Another in vitro time-kill study evaluated meropenem and polymyxin B, amikacin and meropenem, amikacin and rifampin, and amikacin and polymyxin B combinations in 22 clinical XDR *P. aeruginosa* isolates [30]. All dual combinations showed minimal bactericidal activity, while triple combinations of amikacin, rifampin, and polymyxin B and amikacin, meropenem, and polymyxin B exhibited bactericidal activity against 7/10 and 6/10 isolates, respectively.

Clinical studies evaluating the combination treatment of XDR *P. aeruginosa* infections remain limited. In a retrospective cohort study by Rigatto et al., polymyxin B in combination with an antimicrobial lacking in vitro activity was compared to polymyxin B monotherapy in 101 critically ill patients [20]. All isolates were considered XDR and the respiratory tract was the primary infection site. Most patients in the combination therapy group received carbapenems despite most of the isolate minimum inhibitory concentrations (MICs) exceeding 32 mg/L. The investigators reported that combination therapy was independently associated with lower 30-day mortality (Hazard Ratio (HR) 0.33, 95% CI 0.17–0.64; *p* = 0.001) and all patients (3 out of 18) with *P. aeruginosa* infections who received combination therapy survived. However, this finding should be interpreted carefully as only a small percentage of patients (17.8%) had *P. aeruginosa* infections.

### 3.3. Pandrug-Resistant P. aeruginosa

Among the reported pandrug-resistant (PDR) Gram-negative bacteria, *P. aeruginosa* is one of the most common species, along with *A. baumannii* [31]. Unfortunately, the available data regarding therapeutic options is scarce. A combination treatment of amikacin and meropenem resulted in both clinical and microbiological cure in patients with PDR *P. aeruginosa* ventilator-associated pneumonia (VAP), and the combination was later found to be synergistic in vitro [21]. In a case series of patients with PDR Gram-negative bacteria with most patients having PDR *P. aeruginosa*, combination therapy with colistin and various antimicrobials (carbapenems, third generation cephalosporins, and quinolones) achieved clinical and microbiological cure [22]. The authors suggested that the combinations were potentially synergistic, but synergy was not confirmed by in vitro testing.

## 4. *Klebsiella pneumoniae*

### 4.1. K. pneumoniae Carbapenemase-Producing K. pneumoniae

Several investigations have attempted to leverage a polymyxin antibacterial paired with another agent against KPCKP. Despite the ability of the KPC enzymes to hydrolyze carbapenems, several in vitro investigations have suggested that the addition of meropenem to a polymyxin will achieve more killing of KPCKP than a polymyxin alone [32,33]. A retrospective clinical study by Giannella et al. evaluated the treatment of KPCKP bacteremia and observed that incorporation of high-dose carbapenems into a combination regimen resulted in a lower 14-day mortality (*p* = 0.03); however, antibacterials other than polymyxins (including aminoglycosides and tigecycline) were sometimes used in the antimicrobial combinations (Table 2) [34]. Double combinations that utilize a polymyxin and fosfomycin have also been investigated in vitro against polymyxin-resistant KPCKP, and the combination was able to achieve synergy in time-killing experiments (albeit at high drug concentrations) and a hollow-fiber infection model [35,36]. 

Unlike the potential shown with the carbapenem and polymyxin combinations, carbapenem and tigecycline combination studies have yielded fewer promising results against KPCKP. Numerous in vitro studies failed to observe synergistic killing against KPCKP when a carbapenem was paired with tigecycline [41,48,49], whereas other investigations reported that the use of a carbapenem antagonized the killing of tigecycline [50]. Michail et al. tested tigecycline drug combinations in vivo using a murine thigh infection model and concluded that the addition of meropenem antagonized the activity of tigecycline against three of the eight KPCKP strains that were investigated, whereas the addition of rifampin or gentamicin increased the killing of KPCKP by tigecycline [41]. 

Other dual combinations that utilize at least one carbapenem have also been evaluated against KPCKP. The use of a carbapenem paired with either another carbapenem or a cephalosporin has been shown to potentially synergize against KPCKP in vitro [42,51,52,53], and the utility of a double carbapenem regimen has been validated in vivo as well [42]. Moreover, several clinical investigations evaluated the use of ertapenem paired with another carbapenem and found that the combination of two carbapenems may be an effective clinical treatment for infections caused by KPCKP [43,44]; however, there currently are not any randomized clinical trials that compare the use of a double carbapenem regimen with another standard of care for KPCKP infections (such as ceftazidime-avibactam, meropenem-vaborbactam, or imipenem-cilastatin-relebactam) [54]. Carbapenem dual combinations have also shown promise in vitro or in vivo when a carbapenem was paired with either fosfomycin [55] or an aminoglycoside [38].

In addition to dual combinations that are composed of a carbapenem and another agent, several triple combinations that utilize two antibacterials and a carbapenem have been evaluated against KPCKP. Triple combinations of a carbapenem, a polymyxin, and either tigecycline or an aminoglycoside were capable of killing more KPCKP than double combinations in multiple in vitro studies [32,33,49]. Dynamic in vitro models have also suggested that synergy against KPCKP may be achieved with the triple combination of a carbapenem, a polymyxin, and rifampin [39,40]. In contrast to the encouraging results observed in other studies, Del Bono et al. evaluated the activity of meropenem, tigecycline, and either colistin or gentamicin against 19 KPCKP isolates, and meropenem failed to achieve synergistic killing against any of the studied isolates [56].

### 4.2. Oxacillinase-48-Producing K. pneumoniae

Although carbapenem combinations have been investigated extensively against KPCKP, there are less data concerning the use of carbapenem combinations against oxacillinase-48-producing *K. pneumoniae* (OXA-48-KP). Several in vitro studies observed that double combinations of carbapenems were able to achieve synergistic killing against OXA-48-KP [51,57], whereas a separate investigation found that the addition of colistin was necessary for synergistic killing against polymyxin-resistant OXA-48-KP [58]. The combination of a carbapenem and fosfomycin was also shown to achieve additional killing over either agent alone in multiple in vitro studies [59,60]; however, Bakthavatchalam et al. investigated 34 OXA-48-KP isolates in time-killing experiments and determined that meropenem and fosfomycin achieved synergy in only 24% of the isolates [61]. 

### 4.3. New Delhi Metallo-β-Lactamase-Producing K. pneumoniae

In addition to KPCKP and OXA-48-KP, combinations of carbapenems with other antibacterials have also been investigated against New Delhi metallo-β-lactamase producing *K. pneumoniae* (NDMKP). Similar to OXA-48-KP, several in vitro studies observed that a carbapenem and fosfomycin were capable of achieving synergistic killing against NDMKP [46,59,60], whereas the previously described time-killing investigation by Bakthavatchalam et al. only detected synergy against 1/11 NDMKP isolates [61]. The addition of a carbapenem to a polymyxin has also shown promise in vitro [46,58], but one investigation reported that the combination was more likely to achieve synergy against *K. pneumoniae* that expressed NDM only, whereas isolates that co-expressed OXA-48 and NDM enzymes were less susceptible to the combination [46]. Contrary to the encouraging performance of dual carbapenem combinations against KPCKP and OXA-48-KP, the use of two carbapenems failed to consistently achieve synergistic killing against NDMKP in vitro [51,58]. 

## 5. *Acinetobacter baumannii*

### 5.1. Carbapenem-Resistant A. baumannii

The optimal approach to treating an infection caused by *A. baumannii* depends on the susceptibility profile of the offending organism. For relatively drug-susceptible strains, the IDSA recommends the use of a carbapenem or ampicillin-sulbactam [62]. Unfortunately, carbapenem-resistant *A. baumannii* (CRAB) has spread across the globe, and the likeliness of encountering *A. baumannii* that produce oxacillinases that confer carbapenem resistance varies based on the region and patient population [63]. Polymyxins now represent one of the few remaining drug classes that reliably retain activity against CRAB. According to the International Consensus Guidelines for the Optimal Use of Polymyxins, a polymyxin should be combined with an agent that is active against a specific CRAB strain to increase the likeliness of eradicating the pathogen; however, if another agent that is active against the CRAB strain is not available, the panel voted 8 to 7 to weakly recommend that a polymyxin should be used as monotherapy for the management of CRAB [4]. Although newer agents such a cefiderocol may offer another therapeutic option for combatting drug-resistant *A. baumannii*, the potential utility of a polymyxin in combination with a non-active agent has been an area of ongoing research as well [64]. 

Perhaps one of the most extensively studied antibacterial combinations to counter CRAB is the use of a polymyxin and a carbapenem. When evaluated in vitro, the addition of a carbapenem to a polymyxin demonstrated enhanced killing in susceptibility studies, static time-killing experiments, and dynamic models (Table 3) [65,66,67,68,69,70,71,72,73,74,75]. The combination also demonstrated some promise when evaluated in retrospective clinical studies [76]. The use of a carbapenem with a polymyxin was eventually evaluated in a randomized controlled superiority trial that compared colistin monotherapy to the use of colistin with meropenem for the treatment of serious infections caused by carbapenem-resistant Gram-negative bacteria [77]. Over 300 patients with *A. baumannii* infections were included in the study, and a significant difference in the likeness of clinical failure at 14 days was not observed for patients that received colistin monotherapy in comparison to combination therapy (79% versus 73%, RR 0.93, 95% CI 0.83–1.03). A secondary analysis of the study determined that patients infected with colistin-resistant *A. baumannii* had a higher mortality rate if they were treated with colistin and meropenem in comparison to colistin monotherapy (OR 3.065, 95% CI 1.021–9.202) [78]. The strong majority of the *A. baumannii* strains in the aforementioned trial contained a high meropenem MIC of over 8 mg/L, and some clinicians suspect that the combination of a polymyxin and a carbapenem may have utility for treating *A. baumannii* with lower meropenem MICs of 4 or 8 mg/L [79].

Another combination that has been evaluated for the treatment of CRAB is the use of a polymyxin and minocycline. Against *A. baumannii* isolates that were susceptible to both drugs, synergy studies have yielded generally positive results. One susceptibility study observed that synergy between colistin and minocycline in 8.6% of the tested strains [102], whereas a separate investigation found that colistin and minocycline were synergistic against all 14 *A. baumannii* isolates in time-killing experiments [103]. The combination of colistin and minocycline also significantly reduced the bacterial load of *A. baumannii* investigated in a mouse model to a greater extent than either agent alone (*p* < 0.05) [104]. Against *A. baumannii* with some level of minocycline-resistance, the combination of a polymyxin and minocycline has shown mixed results. In a time-killing study that evaluated minocycline in combination with colistin, although minocycline alone resulted in regrowth of the bacteria after 24 h, the combination of minocycline and colistin was synergistic against 12/13 *A. baumannii* isolates that were evaluated [105]. Another study also observed that colistin and minocycline achieved synergy against four minocycline-resistant isolates in time-killing studies, and the combination also improved the survival of mice over colistin alone when one of the isolates was evaluated in vivo (*p* = 0.05) [90]. In contrast, the combination of minocycline and polymyxin B was unable to achieve killing against a minocycline-resistant *A. baumannii* strain during in vitro time-killing experiments and an in vivo neutropenic murine pneumonia model [89]. Another study utilized a dynamic in vitro model to assess the killing of antibacterial combinations against CRAB, and the authors concluded that the triple combination of minocycline, polymyxin B, and sulbactam was the most effective regimen [106].

Lastly, the combination of a polymyxin and sulbactam has been investigated as a therapeutic option against CRAB. Multiple in vitro studies have suggested that the addition of sulbactam to a polymyxin may enhance the killing of *A. baumannii* despite the presence of sulbactam resistance [65,88,102,107,108,109,110], though antagonism has been reported as well [111]. A retrospective study of over 200 patients evaluated various colistin combinations in comparison to colistin alone for the treatment of bacteremia due to CRAB [76]. Of the 214 patients that received combination therapy, 69 patients received colistin and sulbactam, and not only was in-hospital mortality significantly lower for patients that received combination therapy versus colistin alone (52.3% versus 72.2%, *p* = 0.03), but the colistin and sulbactam group had a similar rate of crude mortality among the different combination arms of the study. Unfortunately, two other retrospective studies failed to observe a significant benefit of adding sulbactam to colistin for the treatment of CRAB infections [80,87]. 

### 5.2. Polymyxin-Resistant A. baumannii

Despite the presence of resistance to polymyxins, several investigations have evaluated whether a polymyxin may be combined with rifampin to counter polymyxin-resistant *A. baumannii* (PRAB). Not only has the combination of a polymyxin and rifampin demonstrated synergy against CRAB during in vitro or in vivo experiments [83,112,113,114], but the combination has achieved synergy or sustained killing against PRAB (or *A. baumannii* with polymyxin-resistant subpopulations) as well [115,116,117]. Despite the promising preliminary investigations, a randomized, open-label clinical trial evaluated 210 patients that either received colistin alone or colistin and rifampin for the treatment of XDR *A. baumannii*, and no difference in 30-day mortality was observed between the two treatment groups (*p* = 0.95) [91]. In addition, a retrospective analysis of patients that received colistin at the Detroit Medical Center determined that concomitant rifampin increased the likeliness of nephrotoxicity (OR 3.81, 95% CI 1.42–10.2) [118].

The combination of a polymyxin and either minocycline or sulbactam has also been evaluated as a treatment option for PRAB. Against a polymyxin-resistant but minocycline-susceptible *A. baumannii* isolate, the combination of polymyxin B and minocycline achieved synergy in time-killing experiments [119]. The combination of polymyxin B and minocycline also demonstrated in vitro synergy against a collection of 25 PDR *A. baumannii* isolates [120]. Similarly, colistin with sulbactam was able to achieve synergy or additivity against PRAB isolates in multiple in vitro studies [121,122]. Clinical studies are needed to fully characterize the utility of a polymyxin with either minocycline or sulbactam for the treatment of PRAB infections.

In addition to combining a polymyxin with an agent that is traditionally used for *A. baumannii* infections, investigators have also evaluated the use of a polymyxin in combination with a glycopeptide or lipopeptide to combat PRAB. Many in vitro or in vivo studies have suggested that the addition of vancomycin (or teicoplanin) to a polymyxin may achieve synergy against polymyxin-susceptible *A. baumannii* [93,96,123,124,125,126]; however, a retrospective study of 57 patients determined that the simultaneous use of colistin and vancomycin increased the risk of acute kidney injury (55.2% versus 28%, *p* = 0.04) without improving clinical outcomes in patients with CRAB infections [92]. Against PRAB isolates, the combination of colistin and vancomycin achieved in vitro synergy in multiple studies [97,127]. The combination of colistin and daptomycin was also evaluated against *A. baumannii*, and although the combination was synergistic against CRAB in time-killing experiments, the combination was only capable of achieving stasis against PRAB [128].

Finally, triple combinations of various antimicrobials have been examined as a measure of last resort against PRAB. Among several in vitro and in vivo studies, proposed combinations have included: colistin, vancomycin, and a carbapenem [97], colistin, vancomycin, and rifampin [125], and polymyxin B, sulbactam, and minocycline (against CRAB with a suboptimal response to polymyxins) [106]. In a case series of ten intensive care unit patients with VAP caused by PDR *A. baumannii*, the use of ampicillin-sulbactam, tigecycline, and colistin resulted in a favorable clinical outcome in 9/10 patients and microbiological eradication in 7/10 patients [129]. The combination of ampicillin-sulbactam, a polymyxin, and a carbapenem was capable of killing PRAB in dynamic in vitro models [99,100], and the combination resulted in superior 30-day mortality for seven patients that were treated for PRAB infections in comparison to 10 patients that received an alternative combination (*p* = 0.03) [101]. The use of such triple combinations may eventually become unnecessary if novel antibacterials with activity against PRAB (such as cefiderocol) are developed [64]. 

## 6. *Staphylococcus aureus*

### 6.1. Vancomycin-non-Susceptible S. aureus

The simultaneous use of vancomycin and ceftaroline has been investigated as a potential combination to combat *S. aureus* with attenuated susceptibility to vancomycin. Time-killing experiments conducted by Hutton et al. demonstrated that vancomycin and ceftaroline achieved synergistic killing against multiple *S. aureus* isolates with a vancomycin MIC of 2 mcg/mL [130]. A clinical case series by Gritsenko et al. concluded that 4/5 cases of vancomycin-refractory methicillin-resistant *S. aureus* (MRSA) bacteremia were successfully treated with the combination of vancomycin and ceftaroline [131]. In addition, a retrospective study by Hornak et al. found that the use of vancomycin plus ceftaroline provided a total microbiologic cure rate of 100% for diverse cases of refractory MRSA bacteremia (Table 4) [132]. In contrast to the promising results observed in other investigations, a retrospective clinical study by Ahmad et al. evaluated 30 patients that received combination therapy with vancomycin (or daptomycin) and ceftaroline to clear a persistent MRSA bacteremia [133]. Patients then completed their total duration of therapy by receiving monotherapy with vancomycin (or daptomycin), or the patients completed their course of antibacterials by continuing the initial combination regimen. Despite receiving two antimicrobials, patients in the combination arm of the study had similar rates of bacteremia recurrence (*p* = 0.27), 30-day mortality (*p* = 0.14), and acute kidney injury (*p* = 0.24). 

Other investigations have evaluated the combination of vancomycin and a β-lactam other than ceftaroline, but many of the studies have focused on MRSA and not necessarily *S. aureus* with reduced vancomycin susceptibility. In dynamic time-killing experiments, nafcillin or cefazolin in combination with vancomycin provided additional killing against heteroresistant VISA and VISA strains over vancomycin alone [134,135]. A retrospective cohort study of 80 patients with MRSA bacteremia (median vancomycin MIC of 1–2 mcg/mL) found that patients who received combination therapy of a β-lactam and vancomycin were more likely to achieve microbiological eradication in comparison to patients who received vancomycin alone (*p* = 0.021) [136]. Another open-label clinical trial investigated 60 adults with MRSA bacteremia and found that patients who received combination treatment of vancomycin and flucloxacillin experienced a non-significantly shorter duration of MRSA bacteremia (*p* = 0.06) [144]. A separate open-label randomized clinical trial evaluated 352 patients with MRSA bacteremia and found that the use of vancomycin or daptomycin in combination with a β-lactam resulted in comparable patient outcomes (absolute difference in composite outcomes for combination versus monotherapy was −4.2%; 95% CI–14.3% to 6.0%) [145]. Furthermore, the trial was stopped early because more patients experienced acute kidney injury in the combination arm (13/174) versus the monotherapy group (1/178). 

### 6.2. Daptomycin-non-Susceptible S. aureus

Similar to the combination of vancomycin and ceftaroline, the simultaneous use of daptomycin and ceftaroline has been investigated as a therapeutic option to combat daptomycin-non-susceptible *S. aureus*. An in vitro study by Werth et al. observed that exposing daptomycin-non-susceptible *S. aureus* to the combination of daptomycin and ceftaroline resulted in enhanced bacterial cell depolarization (*p* = 0.03) and killing by human cathelicidin LL37 (*p* < 0.01) in comparison to daptomycin alone [137]. Rose et al. reported that the simultaneous use of daptomycin and ceftaroline was able to clear a MRSA bacteremia that was caused by a daptomycin-non-susceptible *S. aureus* strain [138]. A subsequent in vitro analysis by the same group demonstrated that exposure of the *S. aureus* isolate to daptomycin alone resulted in regrowth and the amplification of daptomycin resistance, whereas the addition of ceftaroline to daptomycin resulted in sustained killing without regrowth or the emergence of resistance. Although there have been several retrospective studies that support the use of daptomycin and ceftaroline for the treatment of refractory or complicated MRSA bacteremia [139,146,147], there are a dearth of clinical investigations that specifically address the utility of the combination for treating infections due to daptomycin-non-susceptible *S. aureus*. 

Multiple studies have also investigated the utility of β-lactams other than ceftaroline in combination with daptomycin against *S. aureus*. Several in vitro investigations have suggested that the addition of oxacillin to daptomycin may enhance the killing of daptomycin-non-susceptible MRSA [141,142]. Another in vitro study found that the combination of nafcillin and daptomycin resulted in significantly improved antibacterial activity against *S. aureus* in comparison to daptomycin alone (*p* = 0.002) [140]. Ceftobiprole has also been investigated as a potential partner agent with daptomycin, and the combination resulted in potent synergistic activity against MRSA isolates with varying antimicrobial susceptibilities in vitro [148]. Similar to the combination of daptomycin and ceftaroline, the clinical investigations that evaluated the addition of other β-lactams to daptomycin therapy focused on MRSA bacteremia. Although a retrospective cohort study indicated that combination therapy of daptomycin and a β-lactam may benefit patients with MRSA bacteremia [143], a randomized clinical trial found that the addition of a β-lactam to daptomycin or vancomycin therapy did not improve patient outcomes [145]. 

## 7. Conclusions

In summary, many in vitro and in vivo studies have investigated combinations of antibacterials that utilize at least one agent that is not active against the target pathogen individually. Such combinations have also been investigated sporadically in clinical studies, but definitive recommendations powered by large clinical trials are lacking. In addition, newer single agent options such as ceftolozane-tazobactam, ceftazidime-avibactam, meropenem-vaborbactam, imipenem-cilastatin-relebactam, and cefiderocol are likely preferred for certain drug-resistant pathogens. In cases of emerging drug resistance against newer antibacterials, or in situations in which newer agents are not appropriate, specific combination therapies may have utility despite resistance against one or more of the selection antimicrobials. Polymyxins in combination with agents such as rifampin have shown some promise in vitro against *P. aeruginosa*, but clinical studies are lacking. Combinations that utilize carbapenems and polymyxins may have a role in the treatment of difficult-to-treat *K. pneumoniae*, whereas dual and triple combinations of polymyxins, carbapenems, minocycline, sulbactam, and rifampin are potential options for drug-resistant *A. baumannii*. In situations of vancomycin or daptomycin-resistant MRSA without single agent alternatives, pairing one of the aforementioned drugs with ceftaroline or another synergistic beta-lactam may be an option for salvage therapy. Additional clinical studies are needed to fully define the preferred therapy for difficult-to-treat pathogens that cannot be optimally treated with a single agent. 

## Figures and Tables

**Table 1:** Summary of studies that investigated antibacterial combinations against *P. aeruginosa* that included at least one drug that was inactive individually. Studies are reported in the table if the combination was investigated using a dynamic in vitro model, an in vivo model, or the investigation was completed in the clinic. Susceptibility studies, checkerboard analyses, and static time-killing experiments are not reported in the table due to the preliminary nature of the experiments and the extensive combinatorial permutations that are difficult to summarize succinctly.

Organism	Combination (Inactive Drug) ^^^	Inactive Drug Did Not Improve Bacterial Killing or an In Vivo/Clinical Outcome	Inactive Drug Provided Improvement in Bacterial Killing or an In Vivo/Clinical Outcome
MDR *P. aeruginosa*	(ceftolozane-tazobactam) + amikacin or colistin		In vitro dynamic model [12]
	(meropenem) + (ceftazidime) or (aztreonam)		In vivo larvae model [14]
	(rifampin) + colistin		In vivo murine pneumonia model ^¥^ [15] Retrospective clinical [16]
	(beta-lactams *) + colistin		Retrospective clinical [17]
	(aztreonam) + colistin		In vivo murine thigh model [18]
	(tobramycin) + (imipenem)		In vivo murine thigh model [19]
XDR *P. aeruginosa*	(meropenem) + polymyxin B		Retrospective clinical ** [20]
PDR *P. aeruginosa*	(meropenem) + (amikacin)		Retrospective clinical [21]
	(colistin) + (imipenem/cilastatin) or (meropenem) or (ofloxacin)		Retrospective clinical *** [22]
	(colistin) + (meropenem) + (ofloxacin) + (gentamicin)		Retrospective clinical *** [22]

^^^ Drugs were determined to be inactive if the pathogens’ MIC was above the CLSI breakpoint for susceptibility, if available. Polymyxin MICs of 2 or higher were considered inactive. Rifampin was considered to be inactive against the Gram-negative organisms. ^¥^ Intranasal colistin. * Antipseudomonal cephalosporins; piperacillin/tazobactam; carbapenems. ** Only a small percentage of patients (17.8%) had *P. aeruginosa* infections. A majority of patients (69.9%) in the combination therapy group received meropenem. *** Case reports of individual patients were included in the table due to a lack of clinical studies that investigated PDR *P. aeruginosa*.

**Table 2:** Summary of studies that investigated antibacterial combinations against *K. pneumoniae* that included at least one drug that was inactive individually. Studies are reported in the table if the combination was investigated using a dynamic in vitro model, an in vivo model, or the investigation was completed in the clinic. Susceptibility studies, checkerboard analyses, and static time-killing experiments are not reported in the table due to the preliminary nature of the experiments and the extensive combinatorial permutations that are difficult to summarize succinctly.

Organism	Combination (Inactive Drug) ^^^	Inactive Drug Did Not Improve Bacterial Killing or an In Vivo/Clinical Outcome	Inactive Drug Provided Improvement in Bacterial Killing or an In Vivo/Clinical Outcome
KPCKP	(meropenem) + colistin		In vitro dynamic model [33] In vivo rabbit osteomyelitis model [32]
	(meropenem) + colistin + gentamicin		In vivo rabbit osteomyelitis model [32]
	(meropenem) + polymyxin B + fosfomycin		In vitro dynamic model [37]
	(meropenem) + colistin + tigecycline		In vitro dynamic model [33]
	(meropenem) + colistin and/or tigecycline and/or gentamicin		Retrospective clinical [34]
	(meropenem) + (amikacin)		In vivo murine thigh model [38]
	(meropenem) + (rifampin) + polymyxin B		In vitro dynamic model [39,40]
	(meropenem) + (rifampin) + (polymyxin B)		In vitro dynamic model [39]
	(meropenem) + tigecycline	In vivo murine thigh model [41]	
	(polymyxin B) + fosfomycin		In vitro dynamic model [36]
	(ertapenem) + (meropenem or doripenem)		In vivo murine thigh model [42] Retrospective clinical [43,44]
NDMKP	(meropenem) + tigecycline		In vitro dynamic model [45]
	(meropenem) + fosfomycin + polymyxin B		In vitro dynamic model [37]
	(fosfomycin) + colistin		In vitro dynamic model [46]
	(polymyxin B) + amikacin + aztreonam		In vitro dynamic model [47]

^^^ Drugs were determined to be inactive if the pathogens’ MIC was above the CLSI breakpoint for susceptibility, if available. Polymyxin MICs of 2 or higher were considered inactive. Rifampin was considered to be inactive against the Gram-negative organisms.

**Table 3:** Summary of studies that investigated antibacterial combinations against *A. baumannii* that included at least one drug that was inactive individually. Studies are reported in the table if the combination was investigated using a dynamic in vitro model, an in vivo model, or the investigation was completed in the clinic. Susceptibility studies, checkerboard analyses, and static time-killing experiments are not reported in the table due to the preliminary nature of the experiments and the extensive combinatorial permutations that are difficult to summarize succinctly.

Organism	Combination (Inactive Drug) ^^^	Inactive Drug Did Not Improve Bacterial Killing or an In Vivo/Clinical Outcome	Inactive Drug Provided Improvement in Bacterial Killing or an In Vivo/Clinical Outcome
CRAB	(carbapenem) + a polymyxin	Retrospective clinical [80] Randomized controlled trial [77]	In vitro dynamic model [70,72,73,75] In vivo murine thigh model ^¥^ [81] In vivo murine sepsis model [82] Retrospective clinical [76]
	(doripenem) + tigeycline or amikacin		In vivo murine sepsis model [82]
	(carbapenem) + (rifampin)		In vivo murine sepsis model [83] In vivo murine pneumonia model [84]
	(imipenem) + tobramycin		In vivo murine pneumonia model [85]
	(imipenem) + (sulbactam)	In vivo murine pneumonia model [85]	In vivo murine sepsis model [86] In vivo murine pneumonia model [84]
	(doripenem) + (sulbactam)		In vivo murine sepsis model [82]
	(sulbactam) + a polymyxin	In vivo murine thigh model [81] In vivo murine sepsis model [86] Retrospective clinical [80,87]	In vitro dynamic model [88] Retrospective clinical [76]
	(sulbactam) + tobramycin		In vivo murine pneumonia model [85]
	(minocycline) + a polymyxin	In vivo murine pneumonia model [89]	In vivo murine pneumonia model [90]
	(rifampin) or (fusidic acid) + colistin		In vivo murine thigh model [81]
	(rifampin) + colistin	In vivo murine pneumonia model [84] Randomized clinical trial * [91]	In vivo rabbit meningitis model [84]
	(rifampin) + (sulbactam)	In vivo rabbit meningitis model [84]	In vivo murine pneumonia model [84]
	(glycopeptide) + colistin	Retrospective clinical [92]	In vivo wax worm model [93,94,95]
PRAB	(colistin) + (rifampin)		In vitro dynamic model
	(colistin) + (vancomycin)	In vivo murine pneumonia model ** [96] In vivo wax worm model [97]	In vivo wax worm model [93]
	(colistin) + (vancomycin) + (doripenem)		In vivo wax worm model [97]
	(vancomycin) + (doripenem)		In vivo wax worm model [97]
	(colistin) + (daptomycin)		In vivo murine intraperitoneal infection model [98]
	(ampicillin-sulbactam) + (polymyxin B) + (meropenem)		In vitro dynamic model [99,100] Retrospective clinical [101]

^^^ Drugs were determined to be inactive if the pathogens’ MIC was above the CLSI breakpoint for susceptibility, if available. Polymyxin MICs of 2 or higher were considered inactive. Rifampin was considered to be inactive against the Gram-negative organisms. ^¥^ The addition of meropenem led to synergistic killing if the meropenem MIC was 16 or 32 mg/L, but the addition of meropenem did not improve killing if the meropenem MIC was > 32 mg/L. * The combination of colistin and rifampin did not improve 30-day mortality over colistin alone regardless of the rifampin MIC; however, the addition of rifampin did increase the rate of microbiologic eradication (*p* = 0.034). ** Teicoplanin achieved a statistically non-significant increase in survival in comparison to colistin alone.

**Table 4:** Summary of studies that investigated antibacterial combinations against *S. aureus* that included at least one drug that was inactive individually. Studies are reported in the table if the combination was investigated using a dynamic in vitro model, an in vivo model, or the investigation was completed in the clinic. Susceptibility studies, checkerboard analyses, and static time-killing experiments are not reported in the table due to the preliminary nature of the experiments and the extensive combinatorial permutations that are difficult to summarize succinctly.

Organism	Combination (Inactive Drug) ^^^	Inactive Drug Did Not Improve Bacterial Killing or an In Vivo/Clinical Outcome	Inactive Drug Provided Improvement in Bacterial Killing or an In Vivo/Clinical Outcome
Vancomycin-non-susceptible *S. aureus*	(vancomycin) + ceftaroline	Retrospective clinical [133]	Retrospective clinical [131] Retrospective clinical [132]
	(vancomycin) + (nafcillin)		In vitro dynamic model [134]
	(vancomycin) + (cefazolin)		In vitro dynamic model [135]
	(vancomycin) + (beta-lactams) ^¥^		Retrospective clinical [136]
Daptomycin-non-susceptible *S. aureus*	(daptomycin) + ceftaroline	Retrospective clinical [133]	Retrospective clinical [132] In vitro dynamic model [137] In vitro dynamic model and case report of infective endocarditis [138] Retrospective clinical ^*^ [139]
	(daptomycin) + (oxacillin or nafcillin)		In vitro dynamic model [140] In vivo rabbit infective endocarditis model [141] Retrospective clinical [142]
	(daptomycin) + (beta-lactams)		Retrospective clinical ** [143]

^^^ Drugs were determined to be inactive if the pathogens’ MIC was above the CLSI breakpoint for susceptibility, if available, or if the drug was unable to clear persistent infections. ^¥^ Most patients received piperacillin-tazobactam (68%). * Only a small percentage of patients (15%) had daptomycin-non-susceptible isolates. ** Most patients (97.7%) had isolates with daptomycin MIC ≤ 1 mg/L. Most commonly used beta-lactams in the combination group included cefepime and cefazolin.

## Data Availability

The manuscript is a review paper and did not include novel data.

## References

[1] Fritzenwanker M., Imirzalioglu C., Herold S., Wagenlehner F.M., Zimmer K.P., Chakraborty T. (2018). Treatment options for carbapenem-resistant gram-negative infections. Dtsch. Arztebl. Int..

[2] Doi Y. (2019). Treatment Options for carbapenem-resistant gram-negative bacterial infections. Clin. Infect. Dis..

[3] Centers for Disease Control Biggest Threats and Data. https://www.cdc.gov/drugresistance/biggest-threats.html.

[4] Tsuji B.T., Pogue J.M., Zavascki A.P., Paul M., Daikos G.L., Forrest A., Giacobbe D.R., Viscoli C., Giamarellou H., Karaiskos I. (2019). International Consensus Guidelines for the Optimal Use of the Polymyxins: Endorsed by the American College of Clinical Pharmacy (ACCP), European Society of Clinical Microbiology and Infectious Diseases (ESCMID), Infectious Diseases Society of America (IDSA), International Society for Anti-infective Pharmacology (ISAP), Society of Critical Care Medicine (SCCM), and Society of Infectious Diseases Pharmacists (SIDP). Pharmacotherapy.

[5] Logan L.K., Weinstein R.A. (2017). The epidemiology of carbapenem-resistant enterobacteriaceae: The impact and evolution of a global menace. J. Infect. Dis..

[6] David M.Z., Daum R.S. (2017). Treatment of Staphylococcus aureus infections. Curr. Top. Microbiol. Immunol..

[7] Magiorakos A.P., Srinivasan A., Carey R.B., Carmeli Y., Falagas M.E., Giske C.G., Harbarth S., Hindler J.F., Kahlmeter G., Olsson-Liljequist B. (2012). Multidrug-resistant, extensively drug-resistant and pandrug-resistant bacteria: An international expert proposal for interim standard definitions for acquired resistance. Clin. Microbiol Infect..

[8] Timurkaynak F., Can F., Azap O.K., Demirbilek M., Arslan H., Karaman S.O. (2006). In vitro activities of non-traditional antimicrobials alone or in combination against multidrug-resistant strains of *Pseudomonas aeruginosa* and *Acinetobacter baumannii* isolated from intensive care units. Int J Antimicrob Agents.

[9] Tascini C., Gemignani G., Ferranti S., Tagliaferri E., Leonildi A., Lucarini A., Menichetti F. (2004). Microbiological activity and clinical efficacy of a colistin and rifampin combination in multidrug-resistant *Pseudomonas aeruginosa* infections. J. Chemother..

[10] Oie S., Uematsu T., Sawa A., Mizuno H., Tomita M., Ishida S., Okano Y., Kamiya A. (2003). In vitro effects of combinations of antipseudomonal agents against seven strains of multidrug-resistant *Pseudomonas aeruginosa*. J. Antimicrob. Chemother..

[11] Mullane E.M., Avery L.M., Nicolau D.P. (2020). Comparative Evaluation of the In Vitro Activities of WCK 5222 (Cefepime-Zidebactam) and Combination Antibiotic Therapies against Carbapenem-Resistant *Pseudomonas aeruginosa*. Antimicrob. Agents Chemother..

[12] Rico Caballero V., Almarzoky Abuhussain S., Kuti J.L., Nicolau D.P. (2018). Efficacy of Human-Simulated Exposures of Ceftolozane-Tazobactam Alone and in Combination with Amikacin or Colistin against Multidrug-Resistant *Pseudomonas aeruginosa* in an In Vitro Pharmacodynamic Model. Antimicrob. Agents Chemother..

[13] Monogue M.L., Nicolau D.P. (2018). Antibacterial activity of ceftolozane/tazobactam alone and in combination with other antimicrobial agents against MDR *Pseudomonas aeruginosa*. J. Antimicrob Chemother.

[14] Siriyong T., Murray R.M., Bidgood L.E., Young S.A., Wright F., Parcell B.J., Voravuthikunchai S.P., Coote P.J. (2019). Dual β-lactam combination therapy for multi-drug resistant *Pseudomonas aeruginosa* infection: Enhanced efficacy in vivo and comparison with monotherapies of penicillin-binding protein inhibition. Sci Rep..

[15] Aoki N., Tateda K., Kikuchi Y., Kimura S., Miyazaki C., Ishii Y., Tanabe Y., Gejyo F., Yamaguchi K. (2009). Efficacy of colistin combination therapy in a mouse model of pneumonia caused by multidrug-resistant *Pseudomonas aeruginosa*. J. Antimicrob. Chemother..

[16] Tascini C., Gemignani G., Palumbo F., Leonildi A., Tedeschi A., Lambelet P., Lucarini. A., Piaggesi A., Menichetti F. (2006). Clinical and microbiological efficacy of colistin therapy alone or in combination as treatment for multidrug resistant *Pseudomonas aeruginosa* diabetic foot infections with or without osteomyelitis. J. Chemother..

[17] Ribera A., Benavent E., Lora-Tamayo J., Tubau F., Pedrero S., Cabo X., Ariza J., Murillo O. (2015). Osteoarticular infection caused by MDR *Pseudomonas aeruginosa*: The benefits of combination therapy with colistin plus β-lactams. J. Antimicrob. Chemother..

[18] Yamagishi Y., Hagihara M., Kato H., Hirai J., Nishiyama N., Koizumi Y., Sakanashi D., Suematsu H., Nakai H., Mikamo H. (2017). In vitro and in vivo Pharmacodynamics of Colistin and Aztreonam Alone and in Combination against Multidrug-Resistant *Pseudomonas aeruginosa*. Chemotherapy.

[19] Yadav R., Bulitta J.B., Wang J., Nation R.L., Landersdorfer C.B. (2017). Evaluation of Pharmacokinetic/Pharmacodynamic Model-Based Optimized Combination Regimens against Multidrug-Resistant *Pseudomonas aeruginosa* in a Murine Thigh Infection Model by Using Humanized Dosing Schemes. Antimicrob. Agents Chemother..

[20] Rigatto M.H., Vieira F.J., Antochevis L.C., Behle T.F., Lopes N.T., Zavascki A.P. (2015). Polymyxin B in Combination with Antimicrobials Lacking In Vitro Activity versus Polymyxin B in Monotherapy in Critically Ill Patients with *Acinetobacter baumannii* or *Pseudomonas aeruginosa* Infections. Antimicrob. Agents Chemother..

[21] Mentzelopoulos S.D., Pratikaki M., Platsouka E., Kraniotaki H., Zervakis D., Koutsoukou A., Nanas S., Paniara O., Roussos C., Giamarellos-Bourboulis E. (2007). Prolonged use of carbapenems and colistin predisposes to ventilator-associated pneumonia by pandrug-resistant *Pseudomonas aeruginosa*. Intensive Care Med..

[22] Falagas M.E., Bliziotis I.A., Kasiakou S.K., Samonis G., Athanassopoulou P., Michalopoulos A. (2005). Outcome of infections due to pandrug-resistant (PDR) Gram-negative bacteria. BMC Infect. Dis..

[23] Olsson A., Wistrand-Yuen P., Nielsen E.I., Friberg L.E., Sandegren L., Lagerbäck P., Tängdén T. (2020). Efficacy of Antibiotic Combinations against Multidrug-Resistant *Pseudomonas aeruginosa* in Automated Time-Lapse Microscopy and Static Time-Kill Experiments. Antimicrob. Agents Chemother..

[24] Peña C., Suarez C., Ocampo-Sosa A., Murillas J., Almirante B., Pomar V., Aguilar M., Granados A., Calbo E., Rodríguez-Baño J. (2013). Effect of adequate single-drug vs combination antimicrobial therapy on mortality in *Pseudomonas aeruginosa* bloodstream infections: A post Hoc analysis of a prospective cohort. Clin. Infect. Dis..

[25] Falagas M.E., Rafailidis P.I., Kasiakou S.K., Hatzopoulou P., Michalopoulos A. (2006). Effectiveness and nephrotoxicity of colistin monotherapy vs. colistin-meropenem combination therapy for multidrug-resistant Gram-negative bacterial infections. Clin. Microbiol. Infect..

[26] Tang S.Y., Zhang S.W., Wu J.D., Wu F., Zhang J., Dong J.T., Guo P., Zhang D.L., Yang J.T., Zhang W.J. (2018). Comparison of mono- and combination antibiotic therapy for the treatment of *Pseudomonas aeruginosa* bacteraemia: A cumulative meta-analysis of cohort studies. Exp. Ther. Med..

[27] Schmid A., Wolfensberger A., Nemeth J., Schreiber P.W., Sax H., Kuster S.P. (2019). Monotherapy versus combination therapy for multidrug-resistant Gram-negative infections: Systematic Review and Meta-Analysis. Sci Rep..

[28] Tamma P.D., Aitken S.L., Bonomo R.A., Mathers A.J., van Duin D., Clancy C.J. (2021). Infectious Diseases Society of America Guidance on the Treatment of Extended-Spectrum β-lactamase Producing Enterobacterales (ESBL-E), Carbapenem-Resistant Enterobacterales (CRE), and *Pseudomonas aeruginosa* with Difficult-to-Treat Resistance (DTR-P. aeruginosa). Clin. Infect. Dis..

[29] Lee H., Roh K.H., Hong S.G., Shin H.B., Jeong S.H., Song W., Uh Y., Yong D., Lee K. (2016). In Vitro Synergistic Effects of Antimicrobial Combinations on Extensively Drug-Resistant *Pseudomonas aeruginosa* and *Acinetobacter baumannii* isolates. Ann. Lab. Med..

[30] Lim T.P., Lee W., Tan T.Y., Sasikala S., Teo J., Hsu L.Y., Tan T.T., Syahidah N., Kwa A.L. (2011). Effective antibiotics in combination against extreme drug-resistant *Pseudomonas aeruginosa* with decreased susceptibility to polymyxin B. PLoS ONE.

[31] Karakonstantis S., Kritsotakis E.I., Gikas A. (2020). Pandrug-resistant Gram-negative bacteria: A systematic review of current epidemiology, prognosis and treatment options. J. Antimicrob. Chemother..

[32] Crémieux A.C., Dinh A., Nordmann P., Mouton W., Tattevin P., Ghout I., Jayol A., Aimer O., Gatin L., Verdier M.C. (2019). Efficacy of colistin alone and in various combinations for the treatment of experimental osteomyelitis due to carbapenemase-producing *Klebsiella pneumoniae*. J. Antimicrob. Chemother..

[33] Tsala M., Vourli S., Georgiou P.C., Pournaras S., Daikos G.R.L., Mouton J.W., Meletiadis J. (2019). Triple combination of meropenem, colistin and tigecycline was bactericidal in a dynamic model despite mere additive interactions in chequerboard assays against carbapenemase-producing *Klebsiella pneumoniae* isolates. J. Antimicrob. Chemother..

[34] Giannella M., Trecarichi E.M., Giacobbe D.R., De Rosa F.G., Bassetti M., Bartoloni A., Bartoletti M., Losito A.R., Del Bono V., Corcione S. (2018). Effect of combination therapy containing a high-dose carbapenem on mortality in patients with carbapenem-resistant *Klebsiella pneumoniae* bloodstream infection. Int. J. Antimicrob. Agents.

[35] Zhao M., Bulman Z.P., Lenhard J.R., Satlin M.J., Kreiswirth B.N., Walsh T.J., Marrocco A., Bergen P.J., Nation R.L., Li J. (2017). Pharmacodynamics of colistin and fosfomycin: A ’treasure trove’ combination combats KPC-producing *Klebsiella pneumoniae*. J. Antimicrob. Chemother..

[36] Diep J.K., Sharma R., Ellis-Grosse E.J., Abboud C.S., Rao G.G. (2018). Evaluation of Activity and Emergence of Resistance of Polymyxin B and ZTI-01 (Fosfomycin for Injection) against KPC-Producing *Klebsiella pneumoniae*. Antimicrob. Agents Chemother..

[37] Garcia E., Diep J.K., Sharma R., Hanafin P.O., Abboud C.S., Kaye K.S., Li J., Velkov T., Rao G.G. (2021). Evaluation Strategies for Triple-Drug Combinations against Carbapenemase-Producing Klebsiella Pneumoniae in an In Vitro Hollow-Fiber Infection Model. Clin. Pharmacol. Ther..

[38] Hagihara M., Kato H., Yamashita R., Soda M., Watanabe H., Sakanashi D., Shiota A., Asai N., Koizumi Y., Suematsu H. (2020). In vivo study assessed meropenem and amikacin combination therapy against carbapenem-resistant and carbapenemase-producing Enterobacteriaceae strains. J. Infect. Chemother..

[39] Diep J.K., Jacobs D.M., Sharma R., Covelli J., Bowers D.R., Russo T.A., Rao G.G. (2017). Polymyxin B in Combination with Rifampin and Meropenem against Polymyxin B-Resistant KPC-Producing *Klebsiella pneumoniae*. Antimicrob. Agents Chemother..

[40] Onufrak N.J., Smith N.M., Satlin M.J., Bulitta J.B., Tan X., Holden P.N., Nation R.L., Li J., Forrest A., Tsuji B.T. (2020). In pursuit of the triple crown: Mechanism-based pharmacodynamic modelling for the optimization of three-drug combinations against KPC-producing *Klebsiella pneumoniae*. Clin. Microbiol. Infect. Publ. Eur. Soc. Clin. Microbiol. Infect. Dis..

[41] Michail G., Labrou M., Pitiriga V., Manousaka S., Sakellaridis N., Tsakris A., Pournaras S. (2013). Activity of Tigecycline in combination with Colistin, Meropenem, Rifampin, or Gentamicin against KPC-producing Enterobacteriaceae in a murine thigh infection model. Antimicrob. Agents Chemother..

[42] Bulik C.C., Nicolau D.P. (2011). Double-carbapenem therapy for carbapenemase-producing *Klebsiella pneumoniae*. Antimicrob. Agents Chemother..

[43] De Pascale G., Martucci G., Montini L., Panarello G., Cutuli S.L., Di Carlo D., Di Gravio V., Di Stefano R., Capitanio G., Vallecoccia M.S. (2017). Double carbapenem as a rescue strategy for the treatment of severe carbapenemase-producing *Klebsiella pneumoniae* infections: A two-center, matched case-control study. Crit. Care.

[44] Cancelli F., Oliva A., De Angelis M., Mascellino M.T., Mastroianni C.M., Vullo V. (2018). Role of Double-Carbapenem Regimen in the Treatment of Infections due to Carbapenemase Producing Carbapenem-Resistant Enterobacteriaceae: A Single-Center, Observational Study. BioMed Res. Int..

[45] Lim T.P., Cai Y., Hong Y., Chan E.C., Suranthran S., Teo J.Q., Lee W.H., Tan T.Y., Hsu L.Y., Koh T.H. (2015). In vitro pharmacodynamics of various antibiotics in combination against extensively drug-resistant *Klebsiella pneumoniae*. Antimicrob. Agents Chemother..

[46] Albur M.S., Noel A., Bowker K., MacGowan A. (2015). The combination of colistin and fosfomycin is synergistic against NDM-1-producing Enterobacteriaceae in in vitro pharmacokinetic/pharmacodynamic model experiments. Int. J. Antimicrob. Agents.

[47] Bulman Z.P., Chen L., Walsh T.J., Satlin M.J., Qian Y., Bulitta J.B., Peloquin C.A., Holden P.N., Nation R.L., Li J. (2017). Polymyxin Combinations Combat Escherichia coli Harboring mcr-1 and blaNDM-5: Preparation for a Postantibiotic Era. mBio.

[48] Pournaras S., Vrioni G., Neou E., Dendrinos J., Dimitroulia E., Poulou A., Tsakris A. (2011). Activity of tigecycline alone and in combination with colistin and meropenem against *Klebsiella pneumoniae* carbapenemase (KPC)-producing Enterobacteriaceae strains by time-kill assay. Int. J. Antimicrob. Agents.

[49] Zhou C., Wang Q., Jin L., Wang R., Yin Y., Sun S., Zhang J., Wang H. (2020). In vitro Synergistic Activity of Antimicrobial Combinations Against bla (KPC) and bla (NDM)-Producing Enterobacterales With bla (IMP) or mcr Genes. Front. Microbiol..

[50] Bi S., Yao X., Huang C., Zheng X., Xuan T., Sheng J., Xu K., Zheng B., Yang Q. (2019). Antagonistic effect between tigecycline and meropenem: Successful management of KPC-producing *Klebsiella pneumoniae* infection. Infection.

[51] Poirel L., Kieffer N., Nordmann P. (2016). In vitro evaluation of dual carbapenem combinations against carbapenemase-producing Enterobacteriaceae. J. Antimicrob. Chemother..

[52] Zhang W., Guo Y., Yang Y., Dong D., Zheng Y., Zhu D., Hu F. (2020). Study of In Vitro Synergistic Bactericidal Activity of Dual β-Lactam Antibiotics Against KPC-2-Producing *Klebsiella pneumoniae*. Microb. Drug Resist..

[53] Hagiya H., Aoki K., Akeda Y., Yamamoto N., Shanmugakani R.K., Ishii Y., Tomono K. (2019). In Vitro Effectiveness of Meropenem and Cefmetazole Combination Treatment Against KPC-2-Producing Enterobacteriaceae. Microb. Drug Resist..

[54] Mashni O., Nazer L., Le J. (2019). Critical Review of Double-Carbapenem Therapy for the Treatment of Carbapenemase-Producing *Klebsiella pneumoniae*. Ann. Pharmacother..

[55] Albiero J., Sy S.K., Mazucheli J., Caparroz-Assef S.M., Costa B.B., Alves J.L., Gales A.C., Tognim M.C. (2016). Pharmacodynamic Evaluation of the Potential Clinical Utility of Fosfomycin and Meropenem in Combination Therapy against KPC-2-Producing *Klebsiella pneumoniae*. Antimicrob. Agents Chemother..

[56] Del Bono V., Giacobbe D.R., Marchese A., Parisini A., Fucile C., Coppo E., Marini V., Arena A., Molin A., Martelli A. (2017). Meropenem for treating KPC-producing *Klebsiella pneumoniae* bloodstream infections: Should we get to the PK/PD root of the paradox?. Virulence.

[57] Galani I., Nafplioti K., Chatzikonstantinou M., Souli M. (2018). In vitro evaluation of double-carbapenem combinations against OXA-48-producing *Klebsiella pneumoniae* isolates using time-kill studies. J. Med. Microbiol..

[58] Erdem F., Abulaila A., Aktas Z., Oncul O. (2020). In vitro evaluation of double carbapenem and colistin combinations against OXA-48, NDM carbapenemase-producing colistin-resistant *Klebsiella pneumoniae* strains. Antimicrob. Resist. Infect. Control..

[59] Erturk Sengel B., Altinkanat Gelmez G., Soyletir G., Korten V. (2020). In vitro synergistic activity of fosfomycin in combination with meropenem, amikacin and colistin against OXA-48 and/or NDM-producing *Klebsiella pneumoniae*. J. Chemother..

[60] Evren E., Azap O.K., Colakoglu S., Arslan H. (2013). In vitro activity of fosfomycin in combination with imipenem, meropenem, colistin and tigecycline against OXA 48-positive *Klebsiella pneumoniae* strains. Diagn. Microbiol. Infect. Dis..

[61] Bakthavatchalam Y.D., Shankar A., Muthuirulandi Sethuvel D.P., Asokan K., Kanthan K., Veeraraghavan B. (2020). Synergistic activity of fosfomycin-meropenem and fosfomycin-colistin against carbapenem resistant *Klebsiella pneumoniae*: An in vitro evidence. Future Sci. OA.

[62] Kalil A.C., Metersky M.L., Klompas M., Muscedere J., Sweeney D.A., Palmer L.B., Napolitano L.M., O’Grady N.P., Bartlett J.G., Carratala J. (2016). Management of Adults With Hospital-acquired and Ventilator-associated Pneumonia: 2016 Clinical Practice Guidelines by the Infectious Diseases Society of America and the American Thoracic Society. Clin. Infect. Dis..

[63] Hamidian M., Nigro S.J. (2019). Emergence, molecular mechanisms and global spread of carbapenem-resistant *Acinetobacter baumannii*. Microb. Genom..

[64] Zhanel G.G., Golden A.R., Zelenitsky S., Wiebe K., Lawrence C.K., Adam H.J., Idowu T., Domalaon R., Schweizer F., Zhanel M.A. (2019). Cefiderocol: A Siderophore Cephalosporin with Activity Against Carbapenem-Resistant and Multidrug-Resistant Gram-Negative Bacilli. Drugs.

[65] Laishram S., Anandan S., Devi B.Y., Elakkiya M., Priyanka B., Bhuvaneshwari T., Peter J.V., Subramani K., Balaji V. (2016). Determination of synergy between sulbactam, meropenem and colistin in carbapenem-resistant *Klebsiella pneumoniae* and *Acinetobacter baumannii* isolates and correlation with the molecular mechanism of resistance. J. Chemother..

[66] Li J., Fu Y., Zhang J., Zhao Y., Fan X., Yu L., Wang Y., Zhang X., Li C. (2020). The efficacy of colistin monotherapy versus combination therapy with other antimicrobials against carbapenem-resistant *Acinetobacter baumannii* ST2 isolates. J. Chemother..

[67] Oliva A., Garzoli S., De Angelis M., Marzuillo C., Vullo V., Mastroianni C.M., Ragno R. (2019). In-Vitro Evaluation of Different Antimicrobial Combinations with and without Colistin Against Carbapenem-Resistant Acinetobacter Baumannii. Molecules.

[68] Pankey G.A., Ashcraft D.S. (2009). The detection of synergy between meropenem and polymyxin B against meropenem-resistant *Acinetobacter baumannii* using Etest and time-kill assay. Diagn. Microbiol. Infect. Dis..

[69] Soudeiha M.A.H., Dahdouh E.A., Azar E., Sarkis D.K., Daoud Z. (2017). In vitro Evaluation of the Colistin-Carbapenem Combination in Clinical Isolates of A. baumannii Using the Checkerboard, Etest, and Time-Kill Curve Techniques. Front. Cell. Infect. Microbiol..

[70] Tängdén T., Karvanen M., Friberg L.E., Odenholt I., Cars O. (2017). Assessment of early combination effects of colistin and meropenem against *Pseudomonas aeruginosa* and *Acinetobacter baumannii* in dynamic time-kill experiments. Infect. Dis..

[71] Peck K.R., Kim M.J., Choi J.Y., Kim H.S., Kang C.I., Cho Y.K., Park D.W., Lee H.J., Lee M.S., Ko K.S. (2012). In vitro time-kill studies of antimicrobial agents against blood isolates of imipenem-resistant *Acinetobacter baumannii*, including colistin- or tigecycline-resistant isolates. J. Med. Microbiol..

[72] Rao G.G., Ly N.S., Bulitta J.B., Soon R.L., San Roman M.D., Holden P.N., Landersdorfer C.B., Nation R.L., Li J., Forrest A. (2016). Polymyxin B in combination with doripenem against heteroresistant *Acinetobacter baumannii*: Pharmacodynamics of new dosing strategies. J. Antimicrob. Chemother..

[73] Bian X., Liu X., Chen Y., Chen D., Li J., Zhang J. (2019). Dose Optimization of Colistin Combinations against Carbapenem-Resistant *Acinetobacter baumannii* from Patients with Hospital-Acquired Pneumonia in China by Using an In Vitro Pharmacokinetic/Pharmacodynamic Model. Antimicrob. Agents Chemother..

[74] Lenhard J.R., Gall J.S., Bulitta J.B., Thamlikitkul V., Landersdorfer C.B., Forrest A., Nation R.L., Li J., Tsuji B.T. (2016). Comparative pharmacodynamics of four different carbapenems in combination with polymyxin B against carbapenem-resistant *Acinetobacter baumannii*. Int. J. Antimicrob. Agents.

[75] Lenhard J.R., Bulitta J.B., Connell T.D., King-Lyons N., Landersdorfer C.B., Cheah S.E., Thamlikitkul V., Shin B.S., Rao G., Holden P.N. (2017). High-intensity meropenem combinations with polymyxin B: New strategies to overcome carbapenem resistance in *Acinetobacter baumannii*. J. Antimicrob. Chemother..

[76] Batirel A., Balkan I.I., Karabay O., Agalar C., Akalin S., Alici O., Alp E., Altay F.A., Altin N., Arslan F. (2014). Comparison of colistin-carbapenem, colistin-sulbactam, and colistin plus other antibacterial agents for the treatment of extremely drug-resistant *Acinetobacter baumannii* bloodstream infections. Eur. J. Clin. Microbiol. Infect. Dis. Publ. Eur. Soc. Clin. Microbiol..

[77] Paul M., Daikos G.L., Durante-Mangoni E., Yahav D., Carmeli Y., Benattar Y.D., Skiada A., Andini R., Eliakim-Raz N., Nutman A. (2018). Colistin alone versus colistin plus meropenem for treatment of severe infections caused by carbapenem-resistant Gram-negative bacteria: An open-label, randomised controlled trial. Lancet Infect. Dis..

[78] Dickstein Y., Lellouche J., Ben Dalak Amar M., Schwartz D., Nutman A., Daitch V., Yahav D., Leibovici L., Skiada A., Antoniadou A. (2019). Treatment Outcomes of Colistin- and Carbapenem-resistant *Acinetobacter baumannii* Infections: An Exploratory Subgroup Analysis of a Randomized Clinical Trial. Clin. Infect. Dis.

[79] Butler D.A., Biagi M., Tan X., Qasmieh S., Bulman Z.P., Wenzler E. (2019). Multidrug Resistant *Acinetobacter baumannii*: Resistance by Any Other Name Would Still be Hard to Treat. Curr. Infect. Dis. Rep..

[80] Yilmaz G.R., Guven T., Guner R., Kocak Tufan Z., Izdes S., Tasyaran M.A., Acikgoz Z.C. (2015). Colistin alone or combined with sulbactam or carbapenem against A. baumannii in ventilator-associated pneumonia. J. Infect. Dev. Ctries..

[81] Fan B., Guan J., Wang X., Cong Y. (2016). Activity of Colistin in Combination with Meropenem, Tigecycline, Fosfomycin, Fusidic Acid, Rifampin or Sulbactam against Extensively Drug-Resistant *Acinetobacter baumannii* in a Murine Thigh-Infection Model. PLoS ONE.

[82] Dinc G., Demiraslan H., Elmali F., Ahmed S.S., Alp E., Doganay M. (2015). Antimicrobial efficacy of doripenem and its combinations with sulbactam, amikacin, colistin, tigecycline in experimental sepsis of carbapenem-resistant *Acinetobacter baumannii*. New Microbiol..

[83] Sun Y., Wang L., Li J., Zhao C., Zhao J., Liu M., Wang S., Lu C., Shang G., Jia Y. (2014). Synergistic efficacy of meropenem and rifampicin in a murine model of sepsis caused by multidrug-resistant *Acinetobacter baumannii*. Eur. J. Pharmacol..

[84] Pachón-Ibáñez M.E., Docobo-Pérez F., López-Rojas R., Domínguez-Herrera J., Jiménez-Mejias M.E., García-Curiel A., Pichardo C., Jiménez L., Pachón J. (2010). Efficacy of rifampin and its combinations with imipenem, sulbactam, and colistin in experimental models of infection caused by imipenem-resistant *Acinetobacter baumannii*. Antimicrob. Agents Chemother..

[85] Montero A., Ariza J., Corbella X., Doménech A., Cabellos C., Ayats J., Tubau F., Borraz C., Gudiol F. (2004). Antibiotic combinations for serious infections caused by carbapenem-resistant *Acinetobacter baumannii* in a mouse pneumonia model. J. Antimicrob. Chemother..

[86] Dinc G., Demiraslan H., Elmali F., Ahmed S.S., Metan G., Alp E., Doganay M. (2013). Efficacy of sulbactam and its combination with imipenem, colistin and tigecycline in an experimental model of carbapenem-resistant *Acinetobacter baumannii* sepsis. Chemotherapy.

[87] Kalin G., Alp E., Akin A., Coskun R., Doganay M. (2014). Comparison of colistin and colistin/sulbactam for the treatment of multidrug resistant *Acinetobacter baumannii* ventilator-associated pneumonia. Infection.

[88] Bian X., Liu X., Feng M., Bergen P.J., Li J., Chen Y., Zheng H., Song S., Zhang J. (2021). Enhanced bacterial killing with colistin/sulbactam combination against carbapenem-resistant *Acinetobacter baumannii*. Int. J. Antimicrob. Agents.

[89] Bowers D.R., Cao H., Zhou J., Ledesma K.R., Sun D., Lomovskaya O., Tam V.H. (2015). Assessment of minocycline and polymyxin B combination against *Acinetobacter baumannii*. Antimicrob. Agents Chemother..

[90] Yang Y.S., Lee Y., Tseng K.C., Huang W.C., Chuang M.F., Kuo S.C., Lauderdale T.L., Chen T.L. (2016). In Vivo and In Vitro Efficacy of Minocycline-Based Combination Therapy for Minocycline-Resistant *Acinetobacter baumannii*. Antimicrob. Agents Chemother..

[91] Durante-Mangoni E., Signoriello G., Andini R., Mattei A., De Cristoforo M., Murino P., Bassetti M., Malacarne P., Petrosillo N., Galdieri N. (2013). Colistin and rifampicin compared with colistin alone for the treatment of serious infections due to extensively drug-resistant *Acinetobacter baumannii*: A multicenter, randomized clinical trial. Clin. Infect. Dis..

[92] Garnacho-Montero J., Amaya-Villar R., Gutiérrez-Pizarraya A., Espejo-Gutiérrez de Tena E., Artero-González M.L., Corcia-Palomo Y., Bautista-Paloma J. (2013). Clinical efficacy and safety of the combination of colistin plus vancomycin for the treatment of severe infections caused by carbapenem-resistant. *Acinetobacter baumannii*. Chemotherapy.

[93] Yang H., Lv N., Hu L., Liu Y., Cheng J., Ye Y., Li J. (2016). In vivo activity of vancomycin combined with colistin against multidrug-resistant strains of *Acinetobacter baumannii* in a *Galleria mellonella* model. Infect. Dis..

[94] Hornsey M., Phee L., Longshaw C., Wareham D.W. (2013). In vivo efficacy of telavancin/colistin combination therapy in a *Galleria mellonella* model of *Acinetobacter baumannii* infection. Int. J. Antimicrob. Agents.

[95] Hornsey M., Wareham D.W. (2011). In vivo efficacy of glycopeptide-colistin combination therapies in a *Galleria mellonella* model of *Acinetobacter baumannii* infection. Antimicrob. Agents Chemother..

[96] Sanderink D., Cassisa V., Chenouard R., Mahieu R., Kempf M., Dubée V., Eveillard M. (2019). Colistin-glycopeptide combinations against multidrug-resistant *Acinetobacter baumannii* in a mouse model of pneumonia. Future Microbiol..

[97] O’Hara J.A., Ambe L.A., Casella L.G., Townsend B.M., Pelletier M.R., Ernst R.K., Shanks R.M., Doi Y. (2013). Activities of vancomycin-containing regimens against colistin-resistant *Acinetobacter baumannii* clinical strains. Antimicrob. Agents Chemother..

[98] Poulakou G., Renieris G., Sabrakos L., Zarkotou O., Themeli-Digalaki K., Perivolioti E., Kraniotaki E., Giamarellos-Bourboulis E.J., Zavras N. (2019). Daptomycin as adjunctive treatment for experimental infection by *Acinetobacter baumannii* with resistance to colistin. Int. J. Antimicrob. Agents.

[99] Lenhard J.R., Smith N.M., Bulman Z.P., Tao X., Thamlikitkul V., Shin B.S., Nation R.L., Li J., Bulitta J.B., Tsuji B.T. (2017). High-Dose Ampicillin-Sulbactam Combinations Combat Polymyxin-Resistant *Acinetobacter baumannii* in a Hollow-Fiber Infection Model. Antimicrob. Agents Chemother..

[100] Lenhard J.R., Thamlikitkul V., Silveira F.P., Garonzik S.M., Tao X., Forrest A., Soo Shin B., Kaye K.S., Bulitta J.B., Nation R.L. (2017). Polymyxin-resistant, carbapenem-resistant *Acinetobacter baumannii* is eradicated by a triple combination of agents that lack individual activity. J. Antimicrob. Chemother..

[101] Qureshi Z.A., Hittle L.E., O’Hara J.A., Rivera J.I., Syed A., Shields R.K., Pasculle A.W., Ernst R.K., Doi Y. (2015). Colistin-resistant *Acinetobacter baumannii*: Beyond carbapenem resistance. Clin. Infect. Dis..

[102] Sertcelik A., Baran I., Akinci E., Mumcuoglu I., Bodur H. (2020). Synergistic Activities of Colistin Combinations with Meropenem, Sulbactam, Minocycline, Disodium Fosfomycin, or Vancomycin Against Different Clones of Carbapenem-Resistant *Acinetobacter baumannii* Strains. Microb. Drug Resist..

[103] Liang W., Liu X.F., Huang J., Zhu D.M., Li J., Zhang J. (2011). Activities of colistin- and minocycline-based combinations against extensive drug resistant *Acinetobacter baumannii* isolates from intensive care unit patients. BMC Infect. Dis..

[104] Ku N.S., Lee S.H., Lim Y.S., Choi H., Ahn J.Y., Jeong S.J., Shin S.J., Choi J.Y., Choi Y.H., Yeom J.S. (2019). In vivo efficacy of combination of colistin with fosfomycin or minocycline in a mouse model of multidrug-resistant *Acinetobacter baumannii* pneumonia. Sci. Rep..

[105] Tan T.Y., Ng L.S., Tan E., Huang G. (2007). In vitro effect of minocycline and colistin combinations on imipenem-resistant *Acinetobacter baumannii* clinical isolates. J. Antimicrob. Chemother..

[106] Beganovic M., Daffinee K.E., Luther M.K., LaPlante K.L. (2021). Minocycline Alone and in Combination with Polymyxin B, Meropenem, and Sulbactam against Carbapenem-Susceptible and -Resistant *Acinetobacter baumannii* in an In Vitro Pharmacodynamic Model. Antimicrob. Agents Chemother..

[107] Leelasupasri S., Santimaleeworagun W., Jitwasinkul T. (2018). Antimicrobial Susceptibility among Colistin, Sulbactam, and Fosfomycin and a Synergism Study of Colistin in Combination with Sulbactam or Fosfomycin against Clinical Isolates of Carbapenem-Resistant *Acinetobacter baumannii*. J. Pathog..

[108] Temocin F., Erdinc F.S., Tulek N., Demirelli M., Ertem G., Kinikli S., Koksal E. (2015). Synergistic effects of sulbactam in multi-drug-resistant *Acinetobacter baumannii*. Braz. J. Microbiol..

[109] Wei W., Yang H., Liu Y., Ye Y., Li J. (2016). In vitro synergy of colistin combinations against extensively drug-resistant *Acinetobacter baumannii* producing OXA-23 carbapenemase. J. Chemother..

[110] Marie M.A., Krishnappa L.G., Alzahrani A.J., Mubaraki M.A., Alyousef A.A. (2015). A prospective evaluation of synergistic effect of sulbactam and tazobactam combination with meropenem or colistin against multidrug resistant *Acinetobacter baumannii*. Bosn. J. Basic Med. Sci..

[111] Çetinkol Y., Telli M., Altunçekiç Yıldırım A., Çalgın M.K. (2016). Evaluation of the efficacy of colistin/sulbactam combination on carbapenem-resistant *Acinetobacter baumannii* strains. Mikrobiyol. Bul..

[112] Bai Y., Liu B., Wang T., Cai Y., Liang B., Wang R., Liu Y., Wang J. (2015). In Vitro activities of combinations of rifampin with other antimicrobials against multidrug-resistant *Acinetobacter baumannii*. Antimicrob. Agents Chemother..

[113] Cetin E.S., Tekeli A., Ozseven A.G., Us E., Aridogan B.C. (2013). Determination of in vitro activities of polymyxin B and rifampin in combination with ampicillin/sulbactam or cefoperazone/sulbactam against multidrug-resistant *Acinetobacter baumannii* by the E-test and checkerboard methods. Jpn. J. Infect. Dis..

[114] Lim T.P., Tan T.Y., Lee W., Sasikala S., Tan T.T., Hsu L.Y., Kwa A.L. (2011). In-vitro activity of polymyxin B, rifampicin, tigecycline alone and in combination against carbapenem-resistant *Acinetobacter baumannii* in Singapore. PLoS ONE.

[115] Nordqvist H., Nilsson L.E., Claesson C. (2016). Mutant prevention concentration of colistin alone and in combination with rifampicin for multidrug-resistant *Acinetobacter baumannii*. Eur. J. Clin. Microbiol. Infect. Dis. Off. Publ. Eur. Soc. Clin. Microbiol..

[116] Lee H.J., Bergen P.J., Bulitta J.B., Tsuji B., Forrest A., Nation R.L., Li J. (2013). Synergistic activity of colistin and rifampin combination against multidrug-resistant *Acinetobacter baumannii* in an in vitro pharmacokinetic/pharmacodynamic model. Antimicrob. Agents Chemother..

[117] Bae S., Kim M.C., Park S.J., Kim H.S., Sung H., Kim M.N., Kim S.H., Lee S.O., Choi S.H., Woo J.H. (2016). In Vitro Synergistic Activity of Antimicrobial Agents in Combination against Clinical Isolates of Colistin-Resistant *Acinetobacter baumannii*. Agents Chemother..

[118] Pogue J.M., Lee J., Marchaim D., Yee V., Zhao J.J., Chopra T., Lephart P., Kaye K.S. (2011). Incidence of and risk factors for colistin-associated nephrotoxicity in a large academic health system. Clin. Infect. Dis..

[119] Aranzana-Climent V., Buyck J.M., Smani Y., Pachón-Diaz J., Marchand S., Couet W., Grégoire N. (2020). Semi-mechanistic PK/PD modelling of combined polymyxin B and minocycline against a polymyxin-resistant strain of *Acinetobacter baumannii*. Clin. Microbiol. Infect. Publ. Eur. Soc. Clin. Microbiol. Infect. Dis..

[120] Zhang Y., Chen F., Sun E., Ma R., Qu C., Ma L. (2013). In vitro antibacterial activity of combinations of fosfomycin, minocycline and polymyxin B on pan-drug-resistant *Acinetobacter baumannii*. Exp. Ther. Med..

[121] Lertsrisatit Y., Santimaleeworagun W., Thunyaharn S., Traipattanakul J. (2017). In vitro activity of colistin mono- and combination therapy against colistin-resistant *Acinetobacter baumannii*, mechanism of resistance, and clinical outcomes of patients infected with colistin-resistant A. baumannii at a Thai university hospital. Infect. Drug Resist..

[122] Menegucci T.C., Fedrigo N.H., Lodi F.G., Albiero J., Nishiyama S.A.B., Mazucheli J., Carrara-Marroni F.E., Voelkner N.M.F., Gong H., Sy S.K.B. (2019). Pharmacodynamic Effects of Sulbactam/Meropenem/Polymyxin-B Combination Against Extremely Drug Resistant *Acinetobacter baumannii* Using Checkerboard Information. Microb. Drug Resist..

[123] Liu B., Liu Y., Di X., Zhang X., Wang R., Bai Y., Wang J. (2014). Colistin and anti-Gram-positive bacterial agents against *Acinetobacter baumannii*. Rev. Soc. Bras. Med. Trop..

[124] Gordon N.C., Png K., Wareham D.W. (2010). Potent synergy and sustained bactericidal activity of a vancomycin-colistin combination versus multidrug-resistant strains of *Acinetobacter baumannii*. Agents Chemother..

[125] Oliva A., Cipolla A., Vullo V., Venditti M., Mastroianni C.M., Falcone M. (2017). Clinical and in vitro efficacy of colistin plus vancomycin and rifampin against colistin-resistant *Acinetobacter baumannii* causing ventilator-associated pneumonia. New Microbiol..

[126] Ceccarelli G., Oliva A., d’Ettorre G., D’Abramo A., Caresta E., Barbara C.S., Mascellino M.T., Papoff P., Moretti C., Vullo V. (2015). The role of vancomycin in addition with colistin and meropenem against colistin-sensitive multidrug resistant *Acinetobacter baumannii* causing severe infections in a Paediatric Intensive Care Unit. BMC Infect. Dis..

[127] Shinohara D.R., Menegucci T.C., Fedrigo N.H., Migliorini L.B., Carrara-Marroni F.E., Maria Dos Anjos M., Cardoso C.L., Nishiyama S.A.B., Tognim M.C.B. (2019). Synergistic activity of polymyxin B combined with vancomycin against carbapenem-resistant and polymyxin-resistant *Acinetobacter baumannii*: First in vitro study. J. Med. Microbiol..

[128] Galani I., Orlandou K., Moraitou H., Petrikkos G., Souli M. (2014). Colistin/daptomycin: An unconventional antimicrobial combination synergistic in vitro against multidrug-resistant *Acinetobacter baumannii*. Int. J. Antimicrob. Agents.

[129] Assimakopoulos S.F., Karamouzos V., Lefkaditi A., Sklavou C., Kolonitsiou F., Christofidou M., Fligou F., Gogos C., Marangos M. (2019). Triple combination therapy with high-dose ampicillin/sulbactam, high-dose tigecycline and colistin in the treatment of ventilator-associated pneumonia caused by pan-drug resistant *Acinetobacter baumannii*: A case series study. Infez. Med..

[130] Hutton M.A., Sundaram A., Perri M.B., Zervos M.J., Herc E.S. (2020). Assessment of invitrosynergy of daptomycin or vancomycin plus ceftaroline for daptomycin non-susceptible Staphylococcus aureus. Diagn. Microbiol. Infect. Dis..

[131] Gritsenko D., Fedorenko M., Ruhe J.J., Altshuler J. (2017). Combination Therapy With Vancomycin and Ceftaroline for Refractory Methicillin-resistant Staphylococcus aureus Bacteremia: A Case Series. Clin. Ther..

[132] Hornak J.P., Anjum S., Reynoso D. (2019). Adjunctive ceftaroline in combination with daptomycin or vancomycin for complicated methicillin-resistant Staphylococcus aureus bacteremia after monotherapy failure. Ther. Adv. Infect. Dis..

[133] Ahmad O., Crawford T.N., Myint T. (2020). Comparing the Outcomes of Ceftaroline Plus Vancomycin or Daptomycin Combination Therapy Versus Monotherapy in Adults with Complicated and Prolonged Methicillin-Resistant Staphylococcus Aureus Bacteremia Initially Treated with Supplemental Ceftaroline. Infect. Dis. Ther..

[134] Leonard S.N. (2012). Synergy between vancomycin and nafcillin against Staphylococcus aureus in an in vitro pharmacokinetic/pharmacodynamic model. PLoS ONE.

[135] Hagihara M., Wiskirchen D.E., Kuti J.L., Nicolau D.P. (2012). In vitro pharmacodynamics of vancomycin and cefazolin alone and in combination against methicillin-resistant Staphylococcus aureus. Antimicrob. Agents Chemother..

[136] Dilworth T.J., Ibrahim O., Hall P., Sliwinski J., Walraven C., Mercier R.C. (2014). β-Lactams enhance vancomycin activity against methicillin-resistant Staphylococcus aureus bacteremia compared to vancomycin alone. Antimicrob. Agents Chemother..

[137] Werth B.J., Sakoulas G., Rose W.E., Pogliano J., Tewhey R., Rybak M.J. (2013). Ceftaroline increases membrane binding and enhances the activity of daptomycin against daptomycin-nonsusceptible vancomycin-intermediate Staphylococcus aureus in a pharmacokinetic/pharmacodynamic model. Antimicrob. Agents Chemother..

[138] Rose W.E., Schulz L.T., Andes D., Striker R., Berti A.D., Hutson P.R., Shukla S.K. (2012). Addition of ceftaroline to daptomycin after emergence of daptomycin-nonsusceptible Staphylococcus aureus during therapy improves antibacterial activity. Antimicrob. Agents Chemother..

[139] Sakoulas G., Moise P.A., Casapao A.M., Nonejuie P., Olson J., Okumura C.Y., Rybak M.J., Kullar R., Dhand A., Rose W.E. (2014). Antimicrobial salvage therapy for persistent staphylococcal bacteremia using daptomycin plus ceftaroline. Clin. Ther..

[140] Leonard S.N., Rolek K.M. (2013). Evaluation of the combination of daptomycin and nafcillin against vancomycin-intermediate Staphylococcus aureus. J. Antimicrob. Chemother..

[141] Yang S.J., Xiong Y.Q., Boyle-Vavra S., Daum R., Jones T., Bayer A.S. (2010). Daptomycin-oxacillin combinations in treatment of experimental endocarditis caused by daptomycin-nonsusceptible strains of methicillin-resistant Staphylococcus aureus with evolving oxacillin susceptibility (the “seesaw effect”). Antimicrob. Agents Chemother..

[142] Dhand A., Bayer A.S., Pogliano J., Yang S.J., Bolaris M., Nizet V., Wang G., Sakoulas G. (2011). Use of antistaphylococcal beta-lactams to increase daptomycin activity in eradicating persistent bacteremia due to methicillin-resistant Staphylococcus aureus: Role of enhanced daptomycin binding. Clin. Infect. Dis..

[143] Jorgensen S.C.J., Zasowski E.J., Trinh T.D., Lagnf A.M., Bhatia S., Sabagha N., Abdul-Mutakabbir J.C., Alosaimy S., Mynatt R.P., Davis S.L. (2020). Daptomycin Plus β-Lactam Combination Therapy for Methicillin-resistant Staphylococcus aureus Bloodstream Infections: A Retrospective, Comparative Cohort Study. Clin. Infect. Dis..

[144] Davis J.S., Sud A., O’Sullivan M.V.N., Robinson J.O., Ferguson P.E., Foo H., van Hal S.J., Ralph A.P., Howden B.P., Binks P.M. (2016). Combination of Vancomycin and β-Lactam Therapy for Methicillin-Resistant Staphylococcus aureus Bacteremia: A Pilot Multicenter Randomized Controlled Trial. Clin. Infect. Dis..

[145] Tong S.Y.C., Lye D.C., Yahav D., Sud A., Robinson J.O., Nelson J., Archuleta S., Roberts M.A., Cass A., Paterson D.L. (2020). Effect of Vancomycin or Daptomycin With vs Without an Antistaphylococcal β-Lactam on Mortality, Bacteremia, Relapse, or Treatment Failure in Patients With MRSA Bacteremia: A Randomized Clinical Trial. JAMA.

[146] McCreary E.K., Kullar R., Geriak M., Zasowski E.J., Rizvi K., Schulz L.T., Ouellette K., Vasina L., Haddad F., Rybak M.J. (2020). Multicenter Cohort of Patients With Methicillin-Resistant Staphylococcus aureus Bacteremia Receiving Daptomycin Plus Ceftaroline Compared With Other MRSA Treatments. Open Forum Infect. Dis..

[147] Geriak M., Haddad F., Rizvi K., Rose W., Kullar R., LaPlante K., Yu M., Vasina L., Ouellette K., Zervos M. (2019). Clinical Data on Daptomycin plus Ceftaroline versus Standard of Care Monotherapy in the Treatment of Methicillin-Resistant Staphylococcus aureus Bacteremia. Antimicrob. Agents Chemother..

[148] Barber K.E., Werth B.J., Ireland C.E., Stone N.E., Nonejuie P., Sakoulas G., Pogliano J., Rybak M.J. (2014). Potent synergy of ceftobiprole plus daptomycin against multiple strains of Staphylococcus aureus with various resistance phenotypes. J. Antimicrob. Chemother..

